# Comparative Study on Biodegradation of Pure Iron Prepared by Microwave Sintering and Laser Melting

**DOI:** 10.3390/ma15041604

**Published:** 2022-02-21

**Authors:** Yingchao Zhao, Jun Feng, Hui Yu, Wangyang Lin, Xin Li, Yan Tian, Mingchun Zhao

**Affiliations:** 1 College of Mechanical Engineering, University of South China, Hengyang 421001, China; 2020000102@usc.edu.cn (Y.Z.); vander_yu@usc.edu.cn (H.Y.); bruce3lin@usc.edu.cn (W.L.); 2 School of Materials Science and Engineering, Central South University, Changsha 410083, China; lx1122@csu.edu.cn (X.L.); 163111055@csu.edu.cn (Y.T.); mczhao@csu.edu.cn (M.Z.)

**Keywords:** pure iron, biodegradation, biocompatibility, microwave sintering, laser melting

## Abstract

For biodegradable pure iron implants, a higher biodegradation rate is preferred. In this work, we compared the biodegradation of pure iron prepared by microwave sintering and laser melting (designated as MSed Fe and LMed Fe, respectively). The MSed Fe presented a distinct porous structure, while the LMed Fe presented a relatively compact structure without any obvious pores. The biodegradation rate of the MSed Fe was higher than that of the LMed Fe, and their biodegradation rates were higher than that of the as-cast Fe. The biodegradation rates of the MSed Fe and the LMed Fe were approximately 44 and 13 times higher than that of the as cast Fe, respectively. The biodegradation was closely related to the microstructure’s compactness and grain size. Moreover, the MSed Fe and the LMed Fe had satisfactory biocompatibility.

## 1. Introduction

Pure iron is an ideal biodegradable metallic stent material [1,2,3,4], which does not create gas or cause the alkalinization of body fluids during the biodegradation process. It is degraded and absorbable even after the remodeling of the blood vessel. A pure iron implant did not lose its supporting function after an operation, and effectively prevented the elastic retraction of the blood vessel. There was no obvious inflammation in the local tissues and no obvious proliferation of the vascular intima, showing satisfactory histocompatibility [5,6]. However, a stent strut of pure iron can remain in blood vessels for over 1 year [7], which means that the biodegradation rate of a pure iron implant is too slow. An implant remaining in a body for an extended period of time will harm healthy tissues, and may create other symptoms. Though the future use of pure iron as a biomedical implant material is promising, it requires an appropriate biodegradation rate in body fluid environments to meet the requirements for clinical application.

Many studies have indicated that the biodegradation of iron-based alloys depends on the grain size, i.e., a finer grain size leads to a higher biodegradation rate for Fe-based alloys [8,9]. The grain refinement of pure iron provides a promising research direction for improving its biodegradation rate. Microwave sintering and laser melting are new methods for rapid prototyping. Microwave sintering shortens the sintering time and improves element diffusion due to its superior uniform heating and higher microwave heating efficiency compared to conventional sintering [10]. Laser melting cools remarkably fast upon solidification due to its rapid melting rate. This is because the material powders begin rapid in situ cooling in the melt pool [11,12]. Therefore, both microwave sintering and laser melting can easily produce fine grain sizes, and thus are expected to produce pure iron with an increased degradation rate. In addition, microwave sintering and laser melting are net-shape manufacturing techniques, which may produce patient-specific implants with minimal post-processing and shorter lead time compared to conventional manufacturing methods such as casting. So far, both microwave sintering and laser melting have been successfully used to produce iron in biomedical applications [13,14]. However, these studies mostly focused on processing or comparing novel manufacturing methods or alloys to conventionally manufactured pure iron. The differences in the biodegradation of pure iron prepared by microwave sintering (MSed Fe) and pure iron prepared by laser melting (LMed Fe) are not yet understood. It is difficult to directly compare the biodegradation results in different studies because they were each significantly influenced by experimental parameters [15]. Therefore, it is important to directly compare the influence of net-shape manufacturing techniques (microwave sintering and laser melting) on the biodegradation of pure iron. To the best of our knowledge, no work comparing their biodegradation has been previously conducted.

In this study, we used pure iron powder to prepare biodegradable pure iron by microwave sintering and laser melting, separately, and compared the microstructure, hardness, biodegradation, and cytocompatibility of both the MSed Fe and the LMed Fe, paying special attention to their biodegradation.

## 2. Experiments

**Figure 1:**
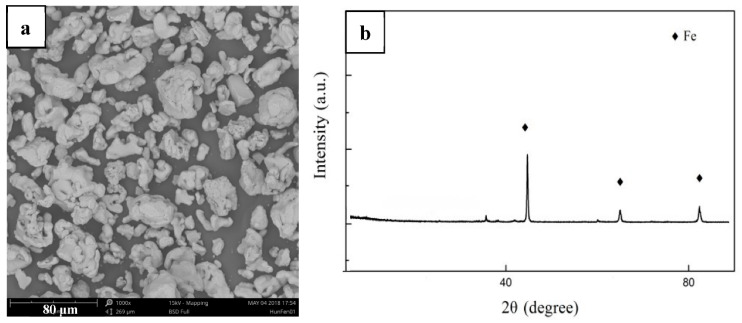
SEM morphology (**a**) and XRD pattern (**b**) of the pure iron powders.

The pure iron powders used in this study were 99.9% pure, containing (by mass) 0.02% Mn, 0.01% Cr, 0.01% Ni, 0.003% C, 0.03% O, 0.004% P, and 0.005% S (purchased from Changsha Tid Metal Materials Co., Ltd., Changsha, China). Figure 1a presents the morphology of the pure iron powders observed by a scanning electron microscope (SEM, ZEISS EVO-18, Jena, Germany), which contained irregular massive clumps, having an average particle diameter of ~30 μm. Figure 1b presents the X-ray diffraction pattern (XRD, with Cu-Kα radiation at 30 mA and 40 kV using 8° min^−1^) of the pure iron powders, in which the three strongest diffraction peaks correspond to α-Fe, and the other small peaks correspond to surface iron oxide, which formed due to minor atmospheric exposure. This indicated that the purity of the pure iron powders were high and without other impurities.

For microwave sintering, cylindrical specimens (Φ10 mm × 5 mm) were obtained by uniaxially compacting pure iron powders for 2 min at 600 MPa, which were demolded by a mold wall lubricant of zinc stearate at 12 mm/min. The obtained specimens were placed in a ceramic crucible microwave acceptor that was set in the glass tube of a microwave sintering furnace (HY-ZG1512, Changsha, China) with 2.45 GHz 4 kW, and was heated by the microwave at 20–1100 °C/min, sintering for 10 min, and then furnace-cooled to room temperature. For laser melting, biodegradable pure iron was fabricated by a self-regulating selective 75 W laser with a 150 µm laser spot size, a scanning speed of 15 mm/s, and a 50 µm hatch spacing and layer thickness. An “S” scan covered the cross section, which interlaced the lower and upper scanning lines. The high-purity Ar atmosphere was used to protect both the microwave sintering and the laser sintering processes.

Microstructures were observed by an optical microscope (OM, ZEISS Axio Vert Al, Jena, Germany), and the densities were measured using the Archimedes method. The Vickers hardness was measured by a microhardness tester (HV-1000, Shanghai, China) under a load of 2.942 N for 15 s. An average hardness was obtained for each specimen by selecting 10 points randomly for a hardness measurement. The pore sizes were measured using direct observation of the cross-section [16]. The density was measured using Archimedes’ principle [17].

The biodegradation was studied using a potentiodynamic polarization test and an immersion test, which were performed in Hank’s solution (containing 8.00 g/L NaCl, 0.40 g/L KCl, 0.10 g/L MgCl_2_·6H_2_O, 0.35 g/L NaHCO_3_, 0.10 g/L MgSO_4_·7H_2_O, 0.14 g/L CaCl_2_, 0.12 g/L Na_2_HPO_4_·12H_2_O, 0.06 g/L KH_2_PO_4_, and 1.00 g/L glucose) with a pH of 7.6 at 37 ± 0.5 °C. More details of the potentiodynamic polarization test and the immersion test have been documented elsewhere [13,14]. Corroded surfaces and topographic maps were observed by a SEM (ZEISS EVO-18, Jena, Germany) and a 3D measuring laser microscope (Barcelona, Spain) after the immersion test, separately. The degradation rate, *P_i_* (mm/year), was calculated from the potentiodynamic polarization test using Equation (1) [13], and the degradation rate, *P_w_* (mm/year), was calculated from immersion test using Equation (2) [14].

(1) $$P_{i}=1.337\times 10^{-2}i_{\mathrm{corr}}$$

(2) $$P_{w}=3.67\left(W_{i}-W_{f}\right)/\left(ATD\right)$$

where *i*_corr_ is the current corrosion density (μA cm^−2^), *W*_i_ is the initial mass before immersion (g), *W*_f_ is the final mass after immersion (g), *A* is the area exposed to the solution (cm^2^), *D* is density (g/cm^3^), and *T* is time (d).

*MG63* cells and ISO 1093-5 indirect contact method were used to detect the cytocompatibility with a fluorescence microscope. More details were documented elsewhere [3]. The *MG63* cell viability was calculated using Equation (3).

(3) $$Cell\;Viability=\left(\frac{OD_{\mathrm{sample}}}{OD_{negative}}\right)\times 100\%$$

where *OD*_sample_ is the sample optical densities and *OD*_negative_ is the negative control.

## 3. Results and Discussion

### 3.1. Microstructure and Hardness

**Figure 2:**
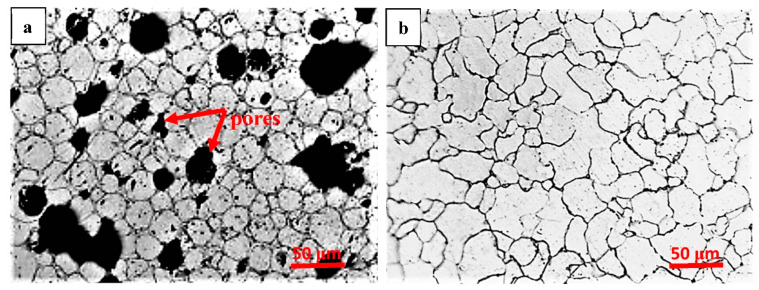
Optical micrographs of both the MSed Fe (**a**) and the LMed Fe (**b**).

Figure 2 shows optical micrographs of MSed Fe and LMed Fe. MSed Fe presented an average ferrite size of ~30 μm, some large pores (~70 μm), and small pores (~10 μm) distributed randomly on its surface. The mechanism of randomly distributed pores on the surface of MSed Fe was ascribed to one or more of the following: (i) power gap, (ii) Kirkendall pores [18], and (iii) impurity evaporation. LMed Fe presented a relatively compact microstructure without any obvious pores, consisting of irregular polygonal ferrite grains, with an average ferrite grain size of ~30 μm. On average, MSed Fe and LMed Fe had smaller ferrite grain sizes than the conventional as cast Fe (with an average ferrite grain size of ~500 μm [19] or ~350 μm [4]). This indicated that a fine ferrite grain size can be produced by microwave sintering or laser melting.

Table 1 lists the density, relative density, and hardness of MSed Fe, LMed Fe, and as-cast Fe. The density of MSed Fe was ~6.85 g/cm^3^, while the density of LMed Fe was higher, at ~7.61 g/cm^3^. The relative density of MSed Fe was ~87.04%, while the relative density of LMed Fe was higher, at ~96.2%. The density and the relative density of the conventional as cast Fe were 7.87 g/cm^3^ and 99.9%, respectively [4]. Therefore, the measured density and relative density indicated that MSed Fe had a porous structure, while LMed Fe had a relatively compact structure, which reflected the microstructure characteristics shown in Figure 2. The hardness of MSed Fe was ~101 HV and that of the LMed Fe was ~113 HV, higher than that of the conventional as cast Fe, which was ~63 HV [19]. This indicated that microwave sintering and laser melting markedly increased hardness. The hardness contribution of the grain refinement was inverse to the grain size, according to the Hall–Petch relation [20]. Thus, hardening was attributed to the grain refinement produced by microwave sintering and laser melting. Furthermore, the hardness of the LMed Fe was higher than that of the MSed Fe, which was attributed to the relatively compact structure in the LMed Fe, rather than the porous structure in the MSed Fe.

### 3.2. Biodegradation

**Figure 3:**
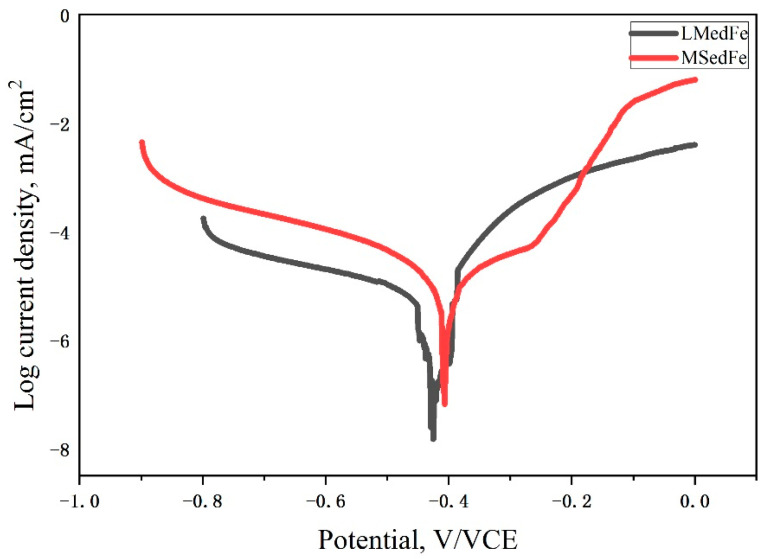
Potentiodynamic polarization curves of MSed Fe and LMed Fe.

Figure 3 presents the potentiodynamic polarization curves of the freshly prepared MSed Fe and LMed Fe. The corrosion potential (*E*_corr_) of MSed Fe was −0.41 V/SCE, while the corrosion potential of LMed Fe was −0.43 V/SCE. The corrosion current density (*i*_corr_) of MSed Fe was 29 μA cm^−2^, and that of LMed Fe was 14.5 μA cm^−2^, which was measured from the linear cathodic branch of the polarization potential curves using Tafel extrapolation [21,22]. Their biodegradation rates were calculated from *i*_corr_ using Equation (1), which are listed in Table 2.

The biodegradation rates of MSed Fe and LMed Fe over 30 days’ immersion is also listed in Table 2, which were calculated from the weight loss using Equation (2). The biodegradation rate from weight loss and that determined from polarization curves gave the same trends, as shown in Table 2, i.e., MSed Fe had a higher biodegradation rate determined from both weight loss or polarization curves compared to LMed Fe. The biodegradation rate was determined from the polarization curves involved in the biodegradation onset, whereas the biodegradation rate determined from the weight loss used the average biodegradation, which included biodegradation for some time after its onset. The biodegradation rate from the weight loss was more realistic, at 0.35 mm/year for MSed Fe, 0.10 mm/year for LMed Fe, and 0.008 mm/year for as-cast Fe [23]. Therefore, MSed Fe had the highest biodegradation rate, the LMed Fe had the second highest, leaving as-cast Fe with the lowest biodegradation rate.

**Figure 4:**
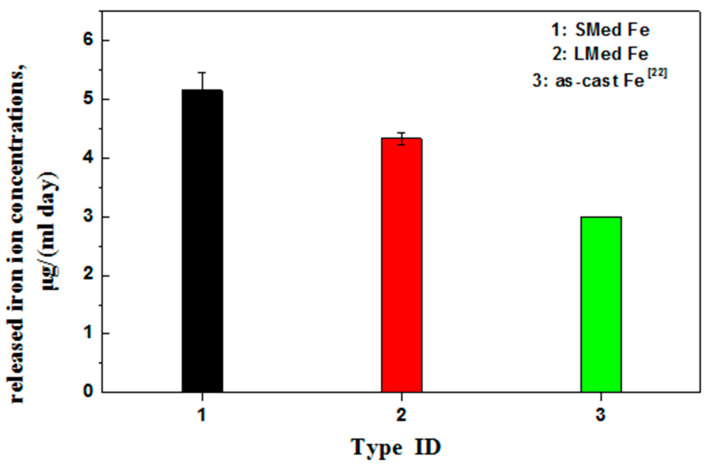
Average daily released iron ion concentrations of MSed Fe, LMed Fe, and as-cast Fe.

Figure 4 presents the average released MSed Fe and LMed Fe concentrations, which were immersed in Hank’s solution for 30 days. The average daily released iron ion concentration was 5.15 μg/(mL day) for MSed Fe and 4.33 μg/(mL day) for LMed Fe. As documented [23], the average daily released iron ion concentration was ~3 μg/(mL day) for as-cast Fe. A higher released iron ion concentration indicates a faster biodegradation rate [24], showing that MSed Fe had a faster biodegradation rate than LMed Fe. Furthermore, these two had faster biodegradation rates than the as-cast Fe. This is consistent with the biodegradation rate determined from the weight loss and polarization curves mentioned above.

**Figure 5:**
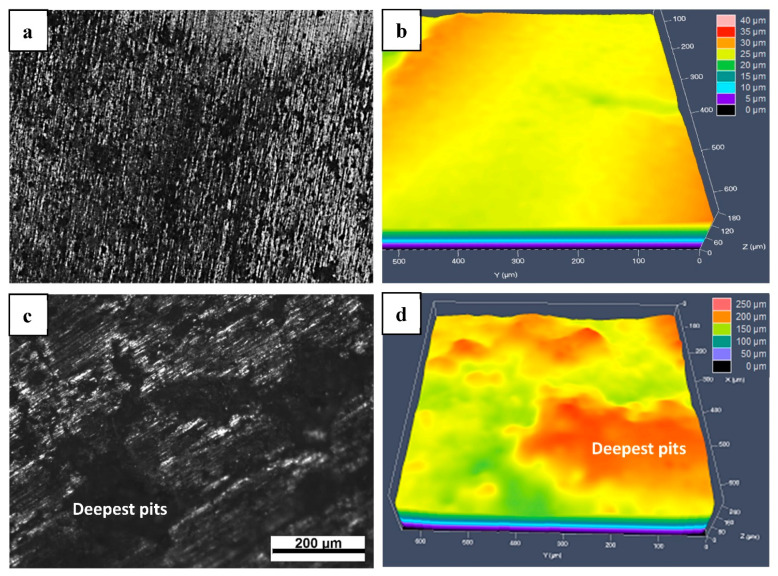
Surface appearances and topographic maps for LMed Fe (**a**,**b**) and MSed Fe (**c**,**d**).

Figure 5 presents the surface appearances and the topographic maps after removing corrosion products for MSed Fe and LMed Fe after 30 days of immersion. Their surface appearances and topographic maps were very different, showing that LMed Fe corroded less. Compared to MSed Fe, LMed Fe showed relatively uniform biodegradation on a macro scale, and the entire corroded surface was homogenously covered by a thin layer of corrosion films. After washing using distilled water, their corresponding topographic maps depicted this difference in biodegradation, in which different colors corresponded to different corrosion depths. The corrosion depth of LMed Fe was shallow, with its deepest corrosion pit at ~30 μm. In contrast, the corrosion depth of SMed Fe was relatively deeper, with its deepest corrosion pit at ~200 μm.

**Figure 6:**
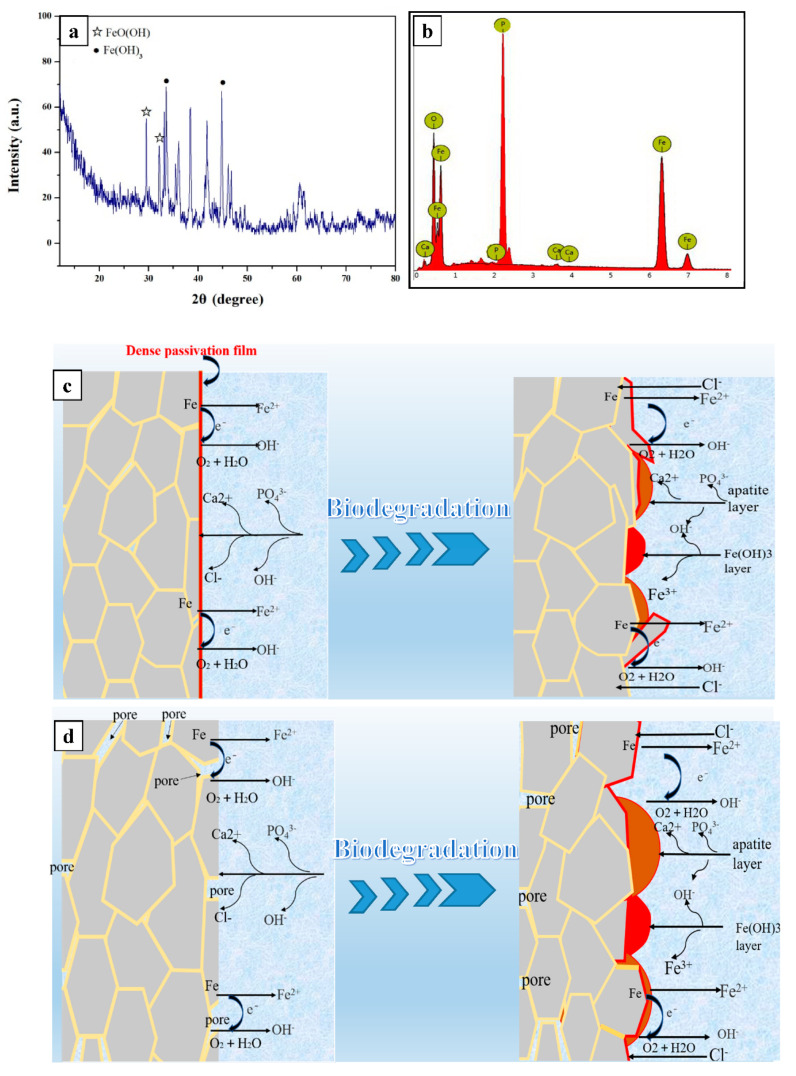
Illustration of biodegradation mechanism: (**a**) XRD; (**b**) EDS results of corrosion products; biodegradation corresponding to (**c**) the compact structure and (**d**) the porous structure.

When metal is implanted into the human body, metal ions are released due to the biodegradation reaction between the metal and the body’s fluids. The biodegradation of the pure iron begins at the grain’s boundaries due to the potential difference between the grain matrix and the grain boundary, and hence, a finer grain size results in a higher biodegradation rate. This is because the finer grains present more grain boundaries, and thus promote biodegradation. The cathodic reaction is: 2 H_2_O + O_2_ + 4 e → 4 OH^−1^, and the anodic reaction is: Fe − 2e → Fe ^2+^. Thus, the following reaction occurs: Fe^2+^+ 2 OH^−1^ → 2 Fe(OH)_2_. The Fe(OH)_2_ is then oxidized into Fe(OH)_3_ due to its instability, followed by the reaction: 4 Fe(OH)_2_ + O_2_ + 2 H_2_O → 4 Fe(OH)_3_. This might be substantiated by the XRD pattern of the biodegradation product of LMed Fe, depicted in Figure 6a, which contained Fe(OH)_3_. When the biodegradation advanced, the Ca/P compounds precipitated on the surface of the hydroxide layer, as demonstrated by the EDS results of LMed Fe. As depicted in Figure 6b, the surface biodegradation products contained O, Fe, Ca and P. In the present work, MSed Fe showed higher biodegradation rates than LMed Fe; these two had higher biodegradation rates than the as-cast Fe. The biodegradation rate was closely related to the compactness of the materials with the porous structure, as substantiated by previous studies [13,25,26]. The porous structure increased the actual area of the contact surface between the materials and the solution. The probable crevice corrosion happened in the porous structure, causing the biodegradation rate to increase. The biodegradation mechanism that was closely related to the compactness of the materials is illustrated in Figure 6c,d where Figure 6c corresponds to the compact structure and Figure 6d corresponds to the porous structure. As described in Figure 2 and Table 1, MSed Fe had a distinct porous structure, while LMed Fe had a relatively compact structure without any obvious pores. Consequentially, the resulting order of the biodegradation rate was as follows: MSed Fe > LMed Fe > as-cast Fe. Moreover, microwave sintering and laser melting caused a fine ferrite grain size. Compared to the as-cast Fe, the grain refinement of the MSed Fe and LMed Fe further promoted this trend.

### 3.3. Biocompatibility

**Figure 7:**
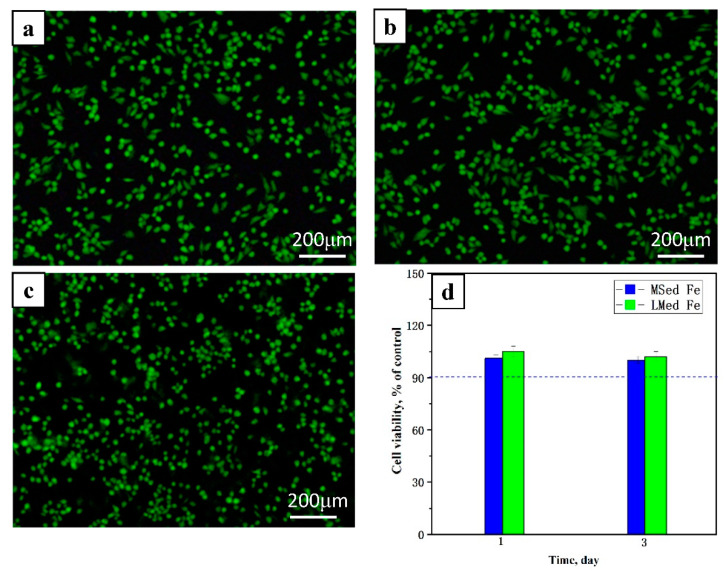
*MG63* cell fluorescence morphologies after 3 day culture: (**a**) extracts, (**b**) MSed Fe, and (**c**) LMed Fe. Cell viability of *MG63* cells in extracts of MSed Fe and LMed Fe after culturing 1 day and 3 days (**d**).

Figure 7 presents the *MG63* cell fluorescence morphologies after culturing for 3 days, in which the living cells are green, and the dead cells are red. Compared to the control group (Figure 7a), the *MG63* cell fluorescence morphology corresponding to the MSed Fe and LMed Fe showed little difference in cell densities (Figure 7b,c). There were no dead cells and numerous living cells were observed in their fluorescence morphologies (Figure 7a–c), indicating normal cell proliferation. *MG63* cell viabilities were higher than 90% in the control group (Figure 7d); therefore, the MSed Fe and the LMed Fe showed no cytotoxicity. Cell viability is a crucial indicator of cytocompatibility; thus, MSed Fe and LMed Fe had satisfactory cytocompatibility.

Metal implant materials release metal ions due to biodegradation, which may be toxic to cells. In the present study, iron ions were released during biodegradation. Iron is an essential element in the human body [27,28], and a normal range of iron ion content is essential for various physiological and metabolic activities. An iron ion concentration of less than 10 μg/mL played a favorable role in endothelial cell metabolic activity, while an iron ion concentration of less than 50 μg/mL hardly inhibited the endothelial cell metabolic activities [29]. As shown in Figure 4, the average daily released iron ion concentration of MSed Fe was the highest, at ~5.15 μg/(mL day), and that of LMed Fe was ~4.33 μg/(mL day), which are far lower than the iron half-maximal inhibitory concentration (IC_50_) [30], and are completely within the safe range of iron ion concentration in the human body. Therefore, the concentration of iron ions did not exceed the cell’s physiological requirements, which was also supported by ion concentrations, cell fluorescence, and cell viability figures, as mentioned above. Furthermore, the biocompatibility of both MSed Fe and LMed Fe was acceptable.

## 4. Conclusions

This comparative study on the biodegradation of pure iron by microwave sintering and laser melting has shown that:

1. MSed Fe presented a distinct porous structure, while LMed Fe presented a relatively compact structure without any obvious pores.
2. MSed Fe had lower a density and hardness than LMed Fe, and both had lower density, but higher hardness than the as-cast Fe.
3. The order of the biodegradation rate was as follows: MSed Fe > LMed Fe > as-cast Fe. That is, the biodegradation rates of MSed Fe and LMed Fe were approximately 44 and 13 times higher than that of as-cast Fe, respectively.
4. Microwave sintering and laser melting were effective methods of increasing the biodegradation rate. The biodegradation behavior was closely related to the microstructure compactness and grain size.
5. MSed Fe and LMed Fe had satisfactory biocompatibility.

## Figures and Tables

**Table 1:** Density and hardness of MSed Fe and LMed Fe.

	MSed Fe	LMed Fe	As-Cast Fe
Density (g/cm^3^)	6.85 ± 0.03	7.61 ± 0.04	7.87 [4]
Relative density (%)	87.04 ± 0.04	96.2 ± 0.02	99.9 [4]
Hardness (HV)	101 ± 2	113 ± 1	63 [19]

**Table 2:** Density and hardness of MSed Fe and LMed Fe.

	MSed Fe	LMed Fe	As-Cast Fe
*i*_corr_, μA/cm^2^	29 ± 0.04	14.5 ± 0.03	4.05 [4]
*P_i_*, mm/year	0.39 ± 0.002	0.20 ± 0.002	-
(*W*_b_ − *W*_a_)/*AT*, mg/(cm^2^ day)	0.65 ± 0.003	0.19 ± 0.001	-
*P_w_*, mm/year	0.35 ± 0.002	0.10 ± 0.001	0.008 [23]

## Data Availability

All data included in this study are available upon request by contact with the first author.
